# Photothermal Responsive Porous Membrane for Treatment of Infected Wound

**DOI:** 10.3390/polym11101679

**Published:** 2019-10-14

**Authors:** Thi Tuong Vy Phan, Thanh-Canh Huynh, Junghwan Oh

**Affiliations:** 1 Center for Advanced Chemistry, Institute of Research and Development, Duy Tan University, 03 Quang Trung, Hai Chau, Danang 550000, Vietnam; phanttuongvy4@duytan.edu.vn; 2 Center for Construction, Mechanics and Materials, Institute of Research and Development, Duy Tan University, 03 Quang Trung, Hai Chau, Danang 550000, Vietnam; huynhthanhcanh@duytan.edu.vn; 3 Center for Marine-Integrated Biomedical Technology, Pukyong National University, Busan 48513, Korea; 4 Department of Biomedical Engineering, Pukyong National University, Busan 48513, Korea

**Keywords:** would healing, palladium nanoparticles, photothermal responsive membrane, chitosan/polyvinyl alcohol membrane, infected wound

## Abstract

Wound infection is a big issue of modern medicine because of multi-drug resistance bacteria; thus, developing an advanced therapy is curial. Photothermal therapy (PTT) is a newly noninvasive strategy that employs PTT agents to transfer near-infrared (NIR) light energy into heat to kill bacterial pathogens. In this work, the PTT agent-containing dressing was developed for the first time to treat the wound infection. Palladium nanoparticles (PdNPs) were chosen as PTT agents because of their high stability, good biocompatibility, excellent photothermal property, and simple-green preparation. With the flexibility and wettability, highly porous membrane chitosan/polyvinyl alcohol (CS/PVA) membrane was chosen as the dressing. The prepared wound dressings exhibited excellent biocompatibility, high porosity, a high degree of swelling, high moisture retention, and high photothermal performance. The treatment of PdNPs loading CS/PVA dressing (CS/PVA/Pd) and laser irradiation killed most of the bacteria in vitro. The proposed PTT agent containing wound dressing introduces a novel strategy for the treatment of wound infection.

## 1. Introduction

Skin is a protective barrier of our body against all environmental factors. When skin is disrupted by burning or cutting, the bacteria can easily occupy and infect the wound bed and lead to infection. With the ever-growing threat of multidrug-resistant bacteria, the infection will not able to be treated with commercial antibiotics. Thus, an infected wound is a serious issue of modern medicine and wound healing has always been an important topic.

Recently, the application of nanomaterials on would healing has raised concerns. Many nanoparticles antimicrobial agents have developed such as silver nanoparticles [1], gold nanoparticles [2], copper and zinc nanoparticles [3,4], iron nanoparticle (FeOOH) [5], etc. However, the slow killing ability and bacteria strain-selective properties are the limitations of antibacterial therapy using nanoparticles.

By converting the near-infrared light (NIR) energy into heat with the assistance of photothermal agents, photothermal therapy (PTT) can be used to treat serious diseases like cancers and bacterial infections [6,7,8]. PTT has become the most potential therapeutic strategy to eliminate multiantibiotic-resistant bacteria owing to its high effectiveness and fast killing ability of bacteria as well as highly selective to treatment tissue as compared with other therapies [7,8,9,10]. Recently, photothermal nanoparticles/hydrogel containing photothermal agents were reported in the field of treatment infected wound and achieved high positively results [11,12,13]. However, the ointments and gel forms of the nanoparticles can be easily wiped from wound exposure sites, therefore reducing their effectiveness of real treatment.

Dressing form can protect the wound from bacteria and the environment factor and promote a suitable environment for wound healing [14]. An ideal wound dressing should include these properties: Nonadhesive, absorb exudate, proving a moist environment for healing, prevents bacterial infection, does not disrupt the healing process and enhances healing without scar formation [14,15].

In this work, the authors have developed novel photothermal responsive 3D porous scaffold as a wound dressing for the treatment of the infected wound. The 3D porous bioscaffolds can mimic the extracellular matrix which can act as a supporting structure for cell adhesion, migration, proliferation, differentiation, and new tissue development [16]. Moreover, the pores structure of 3D scaffolds facilitate nutrient and oxygen diffusion, removing of wastes, and promote wound healing [17]. The photothermal properties of 3D porous scaffold will play the main role in killing bacterial infection of the wound. 

The natural polymers like chitosan, collagen, gelatin, hyaluronic acid are the most attractive candidates for wound healing materials [18,19,20]. The 3D porous scaffolds in this work were fabricated from chitosan (CS) and polyvinyl alcohol (PVA). CS is a natural polysaccharide and has a similarity to glycosaminoglycans in the backbone [21]. CS-based material is widely used in tissue engineering due to its outstanding biological properties such as biocompatibility, biodegradability and also has antibacterial and wound-healing abilities [9,10,22]. However, the poor workability and high brittleness of CS limit its applications [23]. With many excellent properties: Highly hydrophilic properties, excellent mechanical properties, nontoxicity, water-solubility, biocompatibility and biodegradability, PVA can be used to greatly improve flexibility and wettability of CS [23]. The palladium nanoparticles (PdNPs) were chosen as photothermal agents owing to their high stability, good biocompatibility, excellent photothermal property, and simple-green preparation.

The proposed membrane can be used for PTT to eliminate the infection and can protect the wound and create a good environment for wound healing. The PdNPs were embedded in the CS/PVA membrane and tested its killing ability of bacteria with the assistant of the laser. The obtained results evidenced that CS/PVA/PdNPs, which have a high porosity, a high degree of swelling, a high moisture retention, and excellent biocompatibility, could potentially play dual roles in the treatment of an infected wound: (1) killing the bacteria by PTT and (2) keeping the suitable environment for wound healing.

## 2. Materials and Methods

### 2.1. Materials

CS (300 kDa, 90% deacetylation), PVA (89 to 98 kDa), L-ascorbic acid (vitamin C), palladium chloride (PdCl_2_), hydrochloric acid (HCl), DMSO, Luria–Bertani (LB) broth nutrient, and Tryptic Soy Agar were bought from Sigma-Aldrich (St. Louis, MO, USA). Dulbecco′s modified Eagle′s medium (DMEM), fetal bovine serum (FBS), antibiotics, trypsin, and phosphate-buffered saline (PBS) were bought from HyClone (South Logan, UT, USA). 3-(4,5-dimethylthiazol-2-yl)-2,5-diphenyltetrazolium bromide (MTT), acridine orange (AO), and propidium iodide (PI) were also obtained from Sigma-Aldrich (St. Louis, MO, USA). MG-63 osteoblast-like cell line was obtained from the Korean Cell Culture Bank (Seoul, Korea), and Escherichia coli (*E. coli*, ATCC25922) was purchased from the Korean Culture Center of Microorganisms (Seoul, Korea).

### 2.2. One-Pot Synthesis of PdNPs

The one-pot synthesis of PdNPs was adopted from our previous paper [24]. Firstly, 10 mg CS along with 50 mg L-ascorbic acid were added to the glass baker with deposited 15 mL distilled water (DW). The stirring condition was kept for 4 h to completely dissolve CS. Then, 10 mL HPdCl_4_ 0.01 M, which was prepared by adding 44.5 mg PdCl_2_ to 25 mL DW containing 41 µL HCl 37%, was poured to the CS solution. The stirring condition was stopped and leaving the solution for 2 h for the aging of PdNPs. Thereafter, the PdNPs were washed 3 times with PBS to remove all impurity and keeping in vacuum overnight to collect the powder.

### 2.3. Preparation of CS/PVA/Pd Dressing

**Scheme 1:**
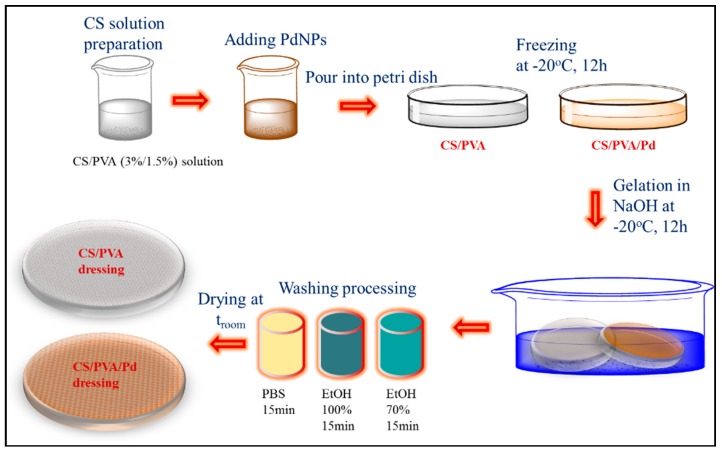
Scheme synthesis of membrane chitosan/polyvinyl alcohol (CS/PVA) and PdNPs loading CS/PVA (CS/PVA/Pd) dressings.

Porous CS/PVA and CS/PVA/Pd dressings were fabricated by the reported method with slight modification [25], illustrating in Scheme 1. CS solution 3 wt % in acetic acid 2% was mixed with 1.5% PVA solution. After getting the homogeneous solution, the PdNPs (5, 10, and 20 ppm) were added to the mixture and stirring condition was kept for 20 min. Thereafter, the final mixture was poured into the Petri dishes (40 mm × 12 mm) and keeping them in the fridge at −20 °C for 12 h. After that, the dressings were transferred to NaOH 3M solution in order to start the gelation process at −20 °C for a further 12 h. Next, the dressings were taken out of the fridge and washed two times with ethanol (70% and 100%, time period: 15 min) and PBS (time period: 15 min). Finally, the dressings were kept at room temperature (*t*_room_) for drying. 

### 2.4. Characterization

Field emission transmission electron microscopic (FE-TEM, JEOL 2010, Tokyo, Japan) images of PdNPs were obtained at an accelerating voltage of 200 kV. Field-emission scanning electron microscope (FE-SEM) images of the membranes were acquired by Hitachi S-2700 microscope (Hitachi, Tokyo, Japan). The membranes were attached to stubs and coated with gold using a sputter coater (Emitech K500X table-top sputter, London, UK) before captured images with FE-SEM. Fourier transform infrared spectroscopy (FTIR, Perkin Elmer Inc., Waltham, MA, USA) was used to analyze the functional groups of dressings. The pyrolysis properties of membranes were analyzed by the thermogravimetric-differential thermal analysis (TG-DTA, Shimadzu DTG-60H, Kyoto, Japan). Fluorescent images of cells and bacteria were captured by a fluorescent microscope (LEICA DMI 3000B, Wetzlar, Germany).

### 2.5. Heating Efficiency Evaluation of PdNPs

The heating efficiency of the PdNPs was studied via a photothermal conversion experiment. Various concentrations (6.25, 12.5, 25, 50, and 60 μg/mL) of PdNPs solution was prepared by dispersing PdNPs powder in PBS, and 1 mL liquor of every concentration was given to a 12-well plate. Then, PdNPs containing well was continuously irradiated under 808 nm-NIR laser (fiber-coupled laser, Changchun New Industries Optoelectronics Technology, Changchun, China) at power density 0.5 W/cm^2^ for 5 min. Thermometer and IR camera were used to capture the temperature changes and the thermal images of the solution, respectively.

### 2.6. Heating Efficiency Evaluation of CS/PVA/Pd Dressing

The heating efficiency of the dressings was evaluated by a photothermal conversion experiment. The CS/PVA as control and CS/PVA/Pd20 as a sample was put on the 6-well plate upon which was deposited 1 mL PBS and continuously irradiated under NIR laser at a power density of 1 W/cm^2^ for 4 min. IR camera was used to capture the thermal images of the dressing.

### 2.7. Porosity Measurement of CS/PVA/Pd Dressing

The porosity of the membrane can be measured by the mass method using ethanol [26]. Briefly, the volumes of ethanol before and after placing the membrane into the glass cylinder were recorded as *V*_1_ and *V*_2_, respectively. After removing the ethanol-absorbed membranes from the cylinder the remaining ethanol volume was *V*_3_. The porosity percentage (*P*) of the membranes was calculated by Equation (1). All samples were triplicate in the experiment.

(1) $$P\;\left(\%\right)=\frac{V_{1}-V_{3}}{V_{2}-V_{3}}\times 100$$

### 2.8. Degree of Swelling and Moisture Retention Capacity of CS/PVA/Pd Dressing

To measure the degree of swelling (*DS*) of the membranes, the same size (dimensions 15 mm × thickness 5 mm) membrane was dipped into 10 mL of DW. After swelling for 2 h, the membrane was taken out and excess water on the surface was wiped off using dry filter paper. Then it was immediately weighted, m_0_ and *m*_w_ are the weight of membrane before and after immersion into water, respectively. The *DS* was calculated by Equation (2) [27]. The experiment was done with three samples of each kind of membrane.

(2) $$DS\;\left(\%\right)=\frac{m_{w}-m_{0}}{m_{0}}\times 100\%$$

To measure the water retention (*WR*) capacity of the membrane, the dry membranes (*m*_0_) with the same size (dimensions 15 mm × thickness 5 mm) were immersed in DW for 2 h. After that, the surface water of swollen membranes was wiped by filter paper. Then, the membranes were placed in open mouth glass Petri at *t*_room_ in 60% relative humidity. The weight of each dressing was determined every 2 h (*m*_t_). The WR was calculated by Equation (3) [28]. The experiment was done with three samples of each kind of membrane.

(3) $$WR\;\left(\%\right)=\frac{m_{t}-m_{0}}{m_{0}}\times 100\%$$

### 2.9. Biocompatible Testing 

Two cytotoxicity assay methods were performed against the MG63 osteosarcoma cell line to test the biocompatible of the prepared dressings including a leachable assay and direct contact assay as follows. All the membranes were after sterilized by radiation under ultraviolet for 30 min. On the leachable assay, 0.2 g sample of each membrane was cut into small pieces (dimensions 2 mm × thickness 2 mm). Then, samples were dipped into DMEM medium and kept in an incubator for 48 h. A density of 5 × 10^3^ cells was seeded in a 96-well plate and was treated with 10 µL of leaching liquor from DMEM containing samples. After incubation for 48 h at 37 °C and 5% CO_2_, the cells were stained by AO/PI to determine the dead and live cells. The cells were incubated with AO (10 μg/mL) and PI (10 μg/mL) at 37 °C for 5 min. The fluorescent imaging was taken by a fluorescent microscope. On the direct contact assay, each thin slice of CS/PVA/Pd membrane was placed in the 6-well plate, MG63 cells were directly seeded on the surface of the membrane and incubated for 24 h. The live/dead cells were examined using an AO/PI double stain. Then, the samples were observed and captured using a fluorescence microscope.

### 2.10. Anti-Bacterial Performance of Photothermal Responsive of CS/PVA/Pd Dressings

The dressing was directly put on the surface of the 1 × 10^8^ CFU/mL bacterial suspension on the 6-well plate. Thereafter, the NIR laser with a power density of 1 W/cm^2^ was used to irradiate each well for 5 min. Then, the bacterial suspension was centrifuged and the supernatant was discarded. The pellets were re-suspended in 1 mL of liquid broth medium and incubated with 10 μL AO (5 mg/mL) + PI (3 mg/mL) at 37 °C for 10 min. The bacterial suspension was again centrifuged at 5000× *g* for 7 min at 4 °C to collect stained bacteria. The centrifugation was repeated 4 times to wash all unincorporated dyes. Finally, the stained bacterial suspension was put on the glass slide and covered with the coverslip and fluorescent images of bacteria were captured for further analysis.

## 3. Result and Discussion 

### 3.1. Characterization of Small PdNPs 

**Figure 1:**
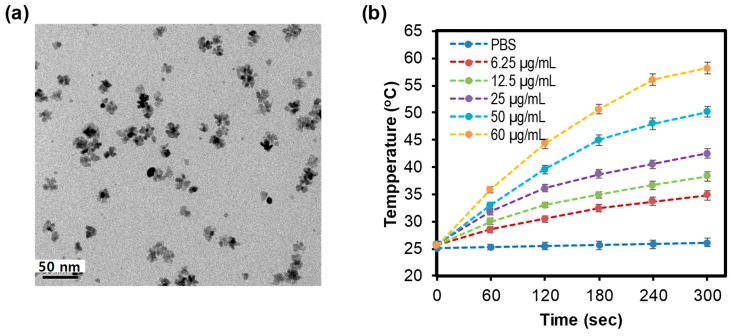
(**a**) Transmission electron microscopic (TEM) of palladiums nanoparticles (PdNPs), (**b**) Heating curve of PdNPs under irradiation (0.5 W/cm^2^, 5 min).

The flower-shaped PdNPs were synthesized by our reported green method [24] and the TEM images of obtained nanoparticles were shown in Figure 1a. The average size of PdNPs was 30.2 ± 17.2 nm. 

The temperature curves of PdNPs solutions with different concentrations, which were recorded via a photothermal conversion experiment, were presented in Figure 1d. The highest concentration of PdNPs solutions (60 μg/mL) rapidly get up to 56.5 °C after 5 min under irradiation (0.5 W/cm^2^); whereas the temperature of lowest concentration one (6.25 μg/mL) only moved up to 33.7 °C. Otherwise, PBS as a control showed a slight change in temperature under the same conditions. The results revealed that the PdNPs have good photothermal properties and can be used as photothermal agents. The gold nanorods were common photothermal agents and their photothermal behavior was well studied. In the previous study, we compared the thermal curve of PdNPs and gold nanorod under the same irradiation condition (808 nm laser at 2  W/cm^2^ power density, 5 min) [29]. The result revealed that the thermal profiles of PdNPs and gold nanorods were quite comparable. Other work also showed that photothermal conversion efficiency of PdNPs was comparable to that of gold nanorods [30].

### 3.2. Characterization Studies of CS/PVA/Pd Dressing

#### 3.2.1. Surface and Morphology of CS/PVA/Pd Dressing

**Scheme 2:**
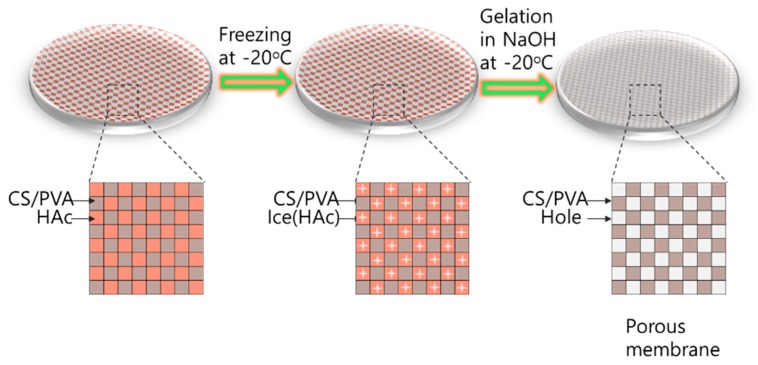
Mechanism of forming porous dressings.

The formation of the porous membrane by the freeze-gelation method can be explained as following [31], illustrating as Scheme 2. During the freezing stage, the CS/PVA and acetic acid were separated from each other from a homogeneous polymer solution. Acetic acid (HAc) was in the ice phase, the CS/PVA was in the rigid phased under −20 °C. When the membrane was placed in NaOH, the gelation happened and acetic acid was removed from a mixed solution; the space occupied by the acetic acid became the pores in the membrane.

**Figure 2:**
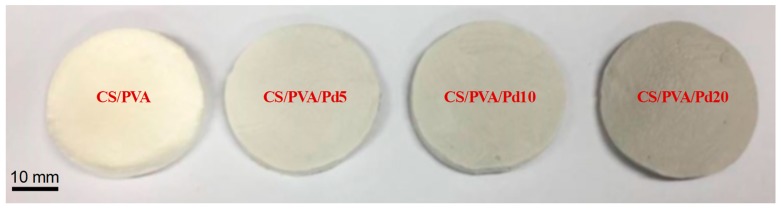
Photograph images of dressings.

The photographic images of the dressings were shown in Figure 2. CS/PVA membrane is in the ivory color, the CS/PVA/Pd membranes have the dark ivory color with dark level corresponding to the concentration of added PdNPs.

**Figure 3:**
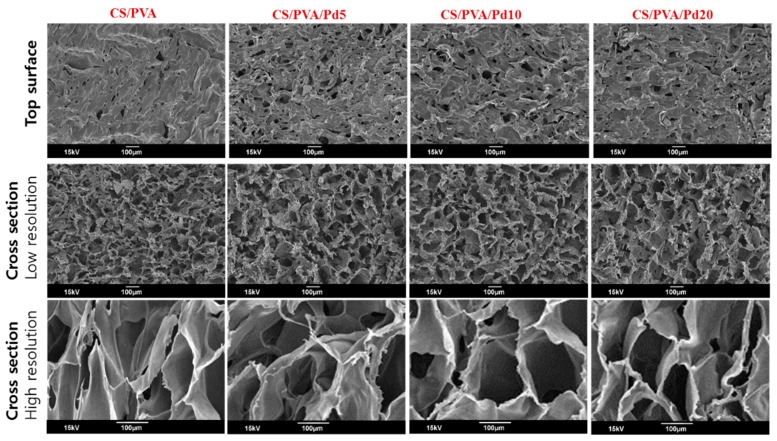
Scanning electron microscope (SEM) of CS/PVA and CS/PVA/Pd dressings.

SEM pictures of the CS/PVA and CS/PVA/Pd were presented in Figure 3. High porosity and large surface area of porous membranes mimic the extracellular matrix that facilitates vascularization and cell migration [26]. As SEM images, the pore diameter and morphology of dressings between 4 groups are not different in size and the average pore diameters about 80–100 µm. This revealed that the cross-linking between CS and PVA mainly determined the morphology and diameter of the pore, a small amount of PdNPs did not cause the change on the pores. 

#### 3.2.2. FT-IR Analysis

**Figure 4:**
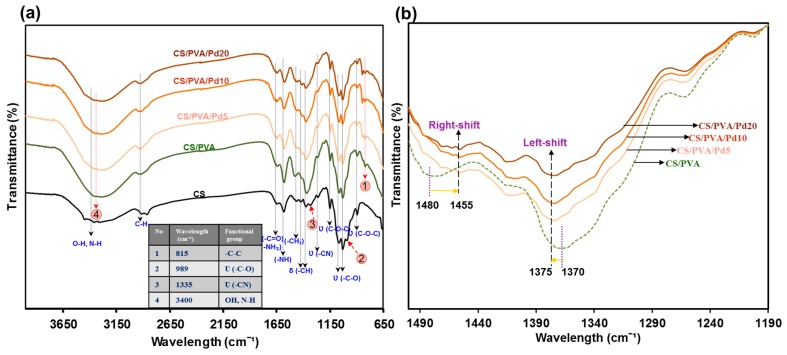
(**a**) Fourier transform infrared spectroscopy (FTIR) of CS/PVA and CS/PVA/PdNPs dressings and (**b**) Shifted peaks on FTIR spectrum of dressings.

Figure 4a displays the spectrum of characteristic peaks obtained from the CS membrane. The CS membrane showed a broadband at 3292–3352 cm^−1^ which corresponded to –OH group, a peak of around 2993 cm^−1^ which corresponded to –CH of the aliphatic groups. The characteristic peaks were located at 1664 and 1041 cm^−1^ which represented the–NH (amide) and C–O–C groups. In the CS/PVA and CS/PVA/Pd membranes spectra, the broadband in the region 3292–3352 cm^−1^ corresponding to the intramolecular hydrogen bonds became more conspicuous. Meanwhile, the peaks at 989 and 1335 cm^−1^ which were associated with –C–O and –CN became ambiguous. A new peak appeared at 815 cm^−1^ which was caused by –C–C– of PVA.

The adding of PdNPs into CS/PVA membrane caused some small effects on the FTIR spectra (Figure 4b) such as the peaks corresponding to CH_2_ wagging and CH_2_ bending had a left-shifted and right-shift and about 5 and 25 cm^−1^ steps, respectively. The peak intensity was reduced when the higher concentration of PdNPs was used.

#### 3.2.3. TGA Analysis

**Figure 5:**
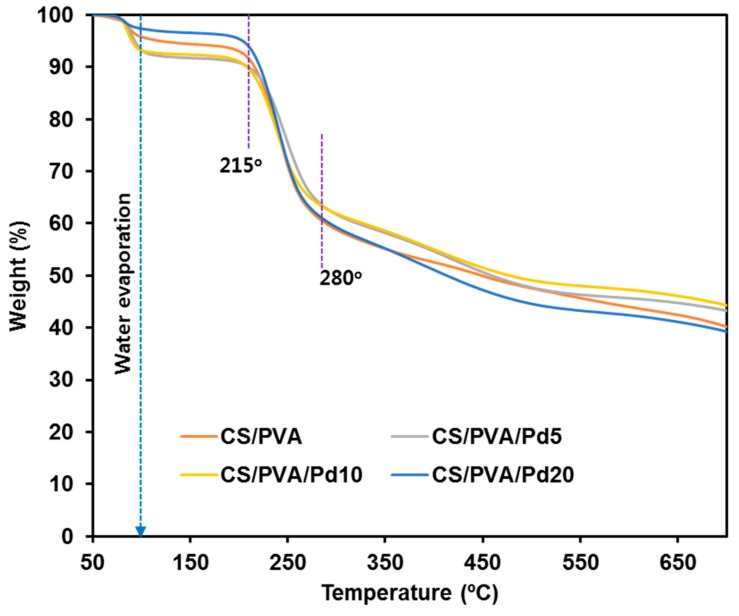
Thermogravimetric analysis (TGA) of CS/PVA and CS/PVA/PdNPs dressings.

Figure 5 showed TGA analysis result of CS/PVA and CS/PVA/Pd membranes. The results show that all of the porous membranes had a thermal degradation temperature range from 215 °C. Under 215 °C, the compositions and properties of membrane were conserved, which totally satisfied the requirement of wound dressing thermal stability. Compared to CS/PVA, CS/PVA/Pd membranes have a slight change in thermal stability when incorporating with PdNPs. In short, a small quantity of PdNPs had no significant effect on the pyrolysis temperature of the membranes.

#### 3.2.4. Porosity 

**Figure 6:**
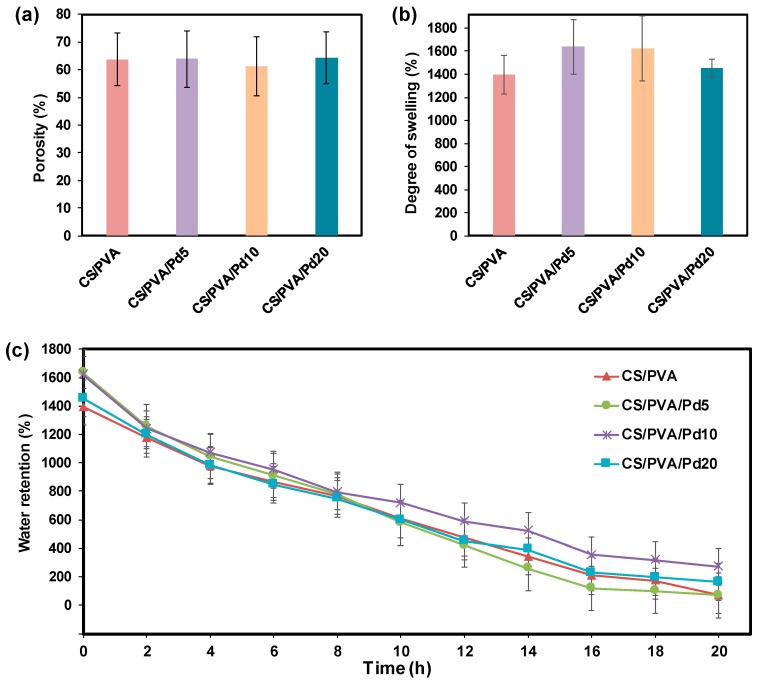
(**a**) Porosity, (**b**) Degree of swelling and (**c**) Water retention of CS/PVA and CS/PVA/Pd dressings.

Figure 6a showed the porosity of the CS/PVA and CS/PVA/Pd membranes. The porosities of all membranes were high and in the range of 62−65%. The high porosity of the membrane helps to absorb exudate from the wound surface effectively and avoid the invasion of microorganisms. Besides that, the nutrients and oxygen can transfer through the interconnected pore networks to the wound site and enable the wound healing process [26]. 

#### 3.2.5. Degree of Swelling and Moisture Retention Capacity

The DS of the membranes was presented in Figure 6b. The DS of the CS/PVA membrane after being fully immersed in water is 1300%, while the DS of CS/PVA/Pd membranes is in the range of 1400−1600%. The adding of PdNPs did not cause significant differences in the DS of CS/PVA and CS/PVA/Pd membranes.

The water retention time of the CS/PVA and CS/PVA/Pd membrane is about 20 h (Figure 6c), which is higher than some previously reported dressing [32,33]. Effective dressing moisture retention helps to reduce clinical infections and scarring [34]. There are no significant differences between the moisture retention of CS/PVA and CS/PVA/Pd dressing.

#### 3.2.6. Heating Evaluation of CS/PVA/Pd Dressing

**Figure 7:**
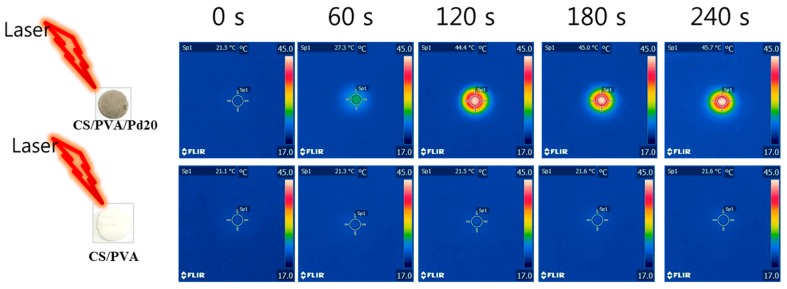
Thermal images of CS/PVA/Pd20 and CS/PVA under laser irradiation (240 s, 1 W/cm^2^).

To test the photothermal behavior of the CS/PVA and CS/PVA/Pd membranes, two samples (CS/PVA and CS/PVA/Pd20) were continuously exposed under the same condition (808 nm laser, 1 W/cm^2^, 240 s). The changes in the temperature of two samples were recorded by a thermal camera (Figure 7). CS/PVA/Pd20 membrane quickly converted the absorbed photon energy into the heat under a power density laser of 1 W/cm^2^ in the short time (240 s), evidencing the good photothermal properties of the prepared membrane. The CS/PVA membrane did not show any change in temperature under the same condition. 

### 3.3. Biocompatibility of CS/PVA/Pd Dressing

**Figure 8:**
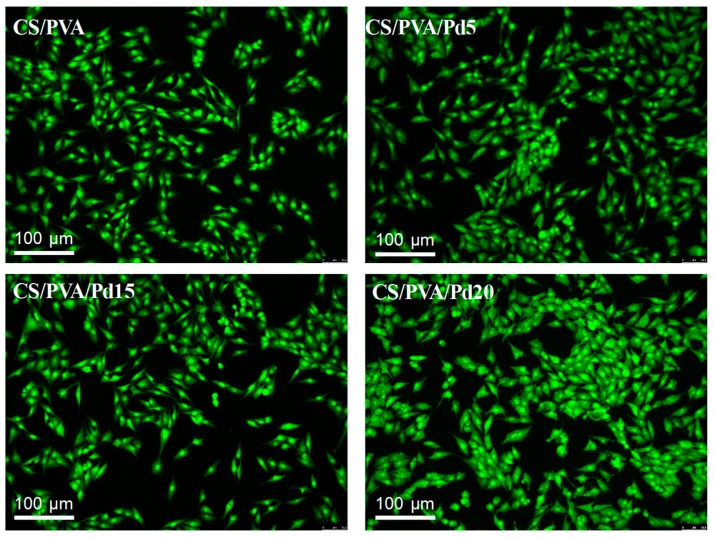
Cytotoxicity assay of CS/PVA and CS/PVA/Pd dressings (incubation time: 48 h).

**Figure 9:**
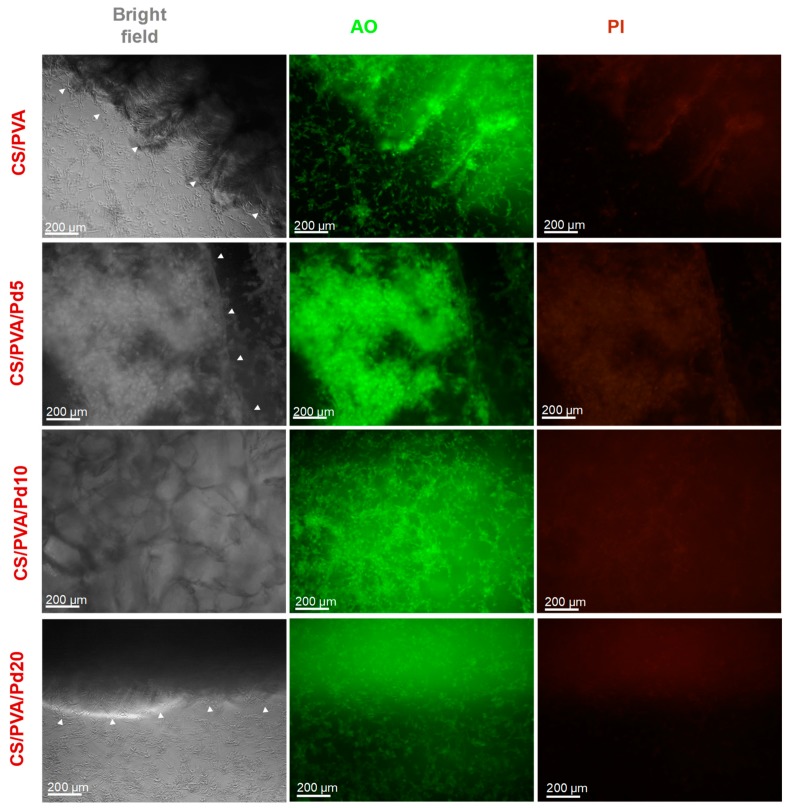
Growing the MG63 cells on the surface of CS/PVA and CS/PVA/Pd dressings. The white head arrows indicate the boundary of the dressings.

Biocompatibility is an important criterion of dressings for wound healing. In the leachable assay, the fluorescent images of AO/PI stained cells (Figure 8) showed that all the cells emitted the green color, indicating no cytotoxicity of the leaching liquor regardless of the incubation time. In the direct contact assay, the cytotoxicity of CS/PVA and CS/PVA/Pd membranes was evaluated by growing directly normal MG63 cell line on the surface of the dressings. By taking the fluorescent imaging of the dressings, the attachment and growth of alive MG63 cells can be observed clearly on the surface of CS/PVA and CS/PVA/Pd membranes (Figure 9). The cytotoxicity test results indicated that the CS/PVA/PdNPs are nontoxic and have a good biocompatibility. The combination of two biocompatible materials including CS/PVA membrane and PdNPs created the biocompatible CS/PVA/PdNPs membrane. The good biocompatible properties of CS/PVA/PdNPs mean it is a promising candidate for human wound dressing. As reported in our previous work, the PdNPs which synthesized by the green method have excellent biocompatibility [24]. The previous report demonstrated that PVA could enhance the flexibility as well as biocompatibility of CS membranes when the ration of CS/PVA = 2/1 [23]. The PVA/CS blend nanofibrous membranes which were fabricated by electrospinning method also showed good biocompatible [35]. Our results have a strong agreement with the reported works. To sum up, the dressings exhibited the good biocompatible properties and are suitable for use in in vivo experiments. 

### 3.4. Anti-Bacterial Performance of CS/PVA/Pd Dressing

**Figure 10:**
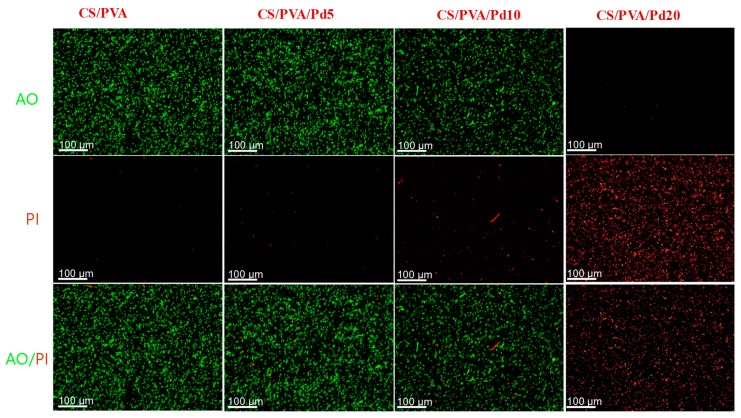
Anti-bacterial performance of CS/PVA and CS/PVA/Pd dressings.

To investigate the in vitro PTT effect of CS/PVA and CS/PVA/Pd dressings, the live and dead assays were performed. The dressing was directly put on the surface of the 1 × 10^8^ CFU/mL *E. coli* bacterial suspension on the 6-wells plate and each well was continuously exposed to 808 nm laser at 1 W/cm^2^ for 10 min. After irradiation, the bacteria were stained with AO/PI to evaluate the bacteria viability. The merged fluorescence image showed that the majority of bacteria on the groups treated with CS/PVA and CS/PVA/Pd5 emitted green fluorescence, indicating negligible dead bacteria. Otherwise, a large number of bacteria on group cells treated CS/PVA/Pd10 showed red fluorescence, indicating that the cells were dead. Most of the bacteria in the CS/PVA/Pd20 treated group emitted red fluorescence, indicating all bacteria were dead (Figure 10). The results evidenced that CS/PVA/Pd20 dressing is effectively killing the bacteria when being exposed to NIR irradiation. 

## 4. Conclusions

In summary, we successfully prepared the CS/PVA/Pd membrane-based PTT in antibacterial therapy of wound infection. The prepared wound dressings exhibited excellent biocompatibility, high porosity, a high degree of swelling, high moisture retention, and high photothermal performance. The PTT in vitro experiment demonstrated the excellent antibacterial ability of prepared dressing nanoparticles via PTT within a very short period of time. To our knowledge, this is the first study reporting on the photothermal responsive membrane as a novel class of wound dressing for NIR photothermal therapy on killing of antibiotic-resistant bacteria. The proposed antibacterial therapy holds a great promise in the treatment of an infected wounds in the future. 

## References

[1] Graham C. (2005). The role of silver in wound healing. Br. J. Nurs..

[2] Leu J.G., Chen S.A., Chen H.M., Wu W.M., Hung C.F., Yao Y.D., Tu C.S., Liang Y.J. (2012). The effects of gold nanoparticles in wound healing with antioxidant epigallocatechin gallate and alpha-lipoic acid. Nanomed. Nanotechnol. Biol. Med..

[3] Tao B., Lin C., Deng Y., Yuan Z., Shen X., Chen M., He Y., Peng Z., Hu Y., Cai K. (2019). Copper-nanoparticle-embedded hydrogel for killing bacteria and promoting wound healing with photothermal therapy. J. Mater. Chem. B.

[4] Varkey A. (2011). Antibacterial properties of some metals and alloys in combating coliforms in contaminated water. Sci. Res. Essays.

[5] Pham D.T.N., Khan F., Phan T.T.V., Park S.-K., Manivasagan P., Oh J., Kim Y.-M. (2019). Biofilm inhibition, modulation of virulence and motility properties by FeOOH nanoparticle in Pseudomonas aeruginosa. Braz. J. Microbiol..

[6] Phan T.T.V., Bui N.Q., Moorthy M.S., Lee K.D., Oh J. (2017). Synthesis and In Vitro Performance of Polypyrrole-Coated Iron-Platinum Nanoparticles for Photothermal Therapy and Photoacoustic Imaging. Nanoscale Res. Lett..

[7] Phan T.T.V., Bharathiraja S., Nguyen V.T., Moorthy M.S., Manivasagan P., Lee K.D., Oh J. (2017). Polypyrrole–methylene blue nanoparticles as a single multifunctional nanoplatform for near-infrared photo-induced therapy and photoacoustic imaging. RSC Adv..

[8] Phan T.T.V., Bui N.Q., Cho S.-W., Bharathiraja S., Manivasagan P., Moorthy M.S., Mondal S., Kim C.-S., Oh J. (2018). Photoacoustic Imaging-Guided Photothermal Therapy with Tumor-Targeting HA-FeOOH@PPy Nanorods. Sci. Rep..

[9] Liang Y., Zhao X., Hu T., Han Y., Guo B. (2019). Mussel-inspired, antibacterial, conductive, antioxidant, injectable composite hydrogel wound dressing to promote the regeneration of infected skin. J. Colloid Interface Sci..

[10] Zhao X., Wu H., Guo B., Dong R., Qiu Y., Ma P.X. (2017). Antibacterial anti-oxidant electroactive injectable hydrogel as self-healing wound dressing with hemostasis and adhesiveness for cutaneous wound healing. Biomaterials.

[11] Zhao Y., Cai Q., Qi W., Jia Y., Xiong T., Fan Z., Liu S., Yang J., Li N., Chang B. (2018). BSA-CuS Nanoparticles for Photothermal Therapy of Diabetic Wound Infection In Vivo. ChemistrySelect.

[12] Zhang L., Wang Y., Wang J., Wang Y., Chen A., Wang C., Mo W., Li Y., Yuan Q., Zhang Y. (2019). Photon-Responsive Antibacterial Nanoplatform for Synergistic Photothermal-/Pharmaco-Therapy of Skin Infection. ACS Appl. Mater. Interfaces.

[13] Liu M., He D., Yang T., Liu W., Mao L., Zhu Y., Wu J., Luo G., Deng J. (2018). An efficient antimicrobial depot for infectious site-targeted chemo-photothermal therapy. J. Nanobiotechnol..

[14] Dhivya S., Padma V.V., Santhini E. (2015). Wound dressings—A review. BioMedicine (Taipei).

[15] Li M., Chen J., Shi M., Zhang H., Ma P.X., Guo B. (2019). Electroactive anti-oxidant polyurethane elastomers with shape memory property as non-adherent wound dressing to enhance wound healing. Chem. Eng. J..

[16] Ou K.-L., Hosseinkhani H. (2014). Development of 3D In Vitro Technology for Medical Applications. Int. J. Mol. Sci..

[17] Bružauskaitė I., Bironaitė D., Bagdonas E., Bernotienė E. (2016). Scaffolds and cells for tissue regeneration: Different scaffold pore sizes-different cell effects. Cytotechnology.

[18] Liang Y., Zhao X., Hu T., Chen B., Yin Z., Ma P.X., Guo B. (2019). Adhesive Hemostatic Conducting Injectable Composite Hydrogels with Sustained Drug Release and Photothermal Antibacterial Activity to Promote Full-Thickness Skin Regeneration during Wound Healing. Small.

[19] Qu J., Zhao X., Liang Y., Zhang T., Ma P.X., Guo B. (2018). Antibacterial adhesive injectable hydrogels with rapid self-healing, extensibility and compressibility as wound dressing for joints skin wound healing. Biomaterials.

[20] Qu J., Zhao X., Liang Y., Xu Y., Ma P.X., Guo B. (2019). Degradable conductive injectable hydrogels as novel antibacterial, anti-oxidant wound dressings for wound healing. Chem. Eng. J..

[21] Yeh H.-Y., Lin J.-C. (2008). Surface characterization and in vitro platelet compatibility study of surface sulfonated chitosan membrane with amino group protection–deprotection strategy. J. Biomater. Sci. Polym. Ed..

[22] Croisier F., Jérôme C. (2013). Chitosan-based biomaterials for tissue engineering. Eur. Polym. J..

[23] Zhuang P.-Y., Li Y.-L., Fan L., Lin J., Hu Q.-L. (2012). Modification of chitosan membrane with poly(vinyl alcohol) and biocompatibility evaluation. Int. J. Biol. Macromol..

[24] Phan T.T.V., Hoang G., Nguyen V.T., Nguyen T.P., Kim H.H., Mondal S., Manivasagan P., Moorthy M.S., Lee K.D., Junghwan O. (2019). Chitosan as a stabilizer and size-control agent for synthesis of porous flower-shaped palladium nanoparticles and their applications on photo-based therapies. Carbohydr. Polym..

[25] Madihally S., Matthew H. (1999). Porous chitosan scafolds for tissue engineering. Biomaterials.

[26] Loh Q.L., Choong C. (2013). Three-dimensional scaffolds for tissue engineering applications: Role of porosity and pore size. Tissue Eng. Part B Rev..

[27] Kumar P.T.S., Abhilash S., Manzoor K., Nair S.V., Tamura H., Jayakumar R. (2010). Preparation and characterization of novel β-chitin/nanosilver composite scaffolds for wound dressing applications. Carbohydr. Polym..

[28] Chandika P., Ko S.C., Oh G.W., Heo S.Y., Nguyen V.T., Jeon Y.J., Lee B., Jang C.H., Kim G., Park W.S. (2015). Fish collagen/alginate/chitooligosaccharides integrated scaffold for skin tissue regeneration application. Int. J. Biol. Macromol..

[29] Bharathiraja S., Bui N.Q., Manivasagan P., Moorthy M.S., Mondal S., Seo H., Phuoc N.T., Vy Phan T.T., Kim H., Lee K.D. (2018). Multimodal tumor-homing chitosan oligosaccharide-coated biocompatible palladium nanoparticles for photo-based imaging and therapy. Sci. Rep..

[30] Xiao J.-W., Fan S.-X., Wang F., Sun L.-D., Zheng X.-Y., Yan C.-H. (2014). Porous Pd nanoparticles with high photothermal conversion efficiency for efficient ablation of cancer cells. Nanoscale.

[31] Ho M.-H., Kuo P.-Y., Hsieh H.-J., Hsien T.-Y., Hou L.-T., Lai J.-Y., Wang D.-M. (2004). Preparation of porous scaffolds by using freeze-extraction and freeze-gelation methods. Biomaterials.

[32] Liu J., Qian Z., Shi Q., Yang S., Wang Q., Liu B., Xu J., Guo X., Liu H. (2017). An asymmetric wettable chitosan–silk fibroin composite dressing with fixed silver nanoparticles for infected wound repair: In Vitro and In Vivo evaluation. RSC Adv..

[33] Li Q., Lu F., Zhou G., Yu K., Lu B., Xiao Y., Dai F., Wu D., Lan G. (2017). Silver Inlaid with Gold Nanoparticle/Chitosan Wound Dressing Enhances Antibacterial Activity and Porosity, and Promotes Wound Healing. Biomacromolecules.

[34] Bolton L.L., Monte K., Pirone L.A. (2000). Moisture and healing: Beyond the jargon. Ostomy/Wound Manag..

[35] Alhosseini S.N., Moztarzadeh F., Mozafari M., Asgari S., Dodel M., Samadikuchaksaraei A., Kargozar S., Jalali N. (2012). Synthesis and characterization of electrospun polyvinyl alcohol nanofibrous scaffolds modified by blending with chitosan for neural tissue engineering. Int. J. Nanomed..

